# Research hotspots and frontiers of neuromodulation technology in the last decade: a visualization analysis based on the Web of Science database

**DOI:** 10.3389/fnhum.2025.1574721

**Published:** 2025-04-11

**Authors:** Yanpei Liu, Qian Zhang, Haoran Zhang, Yun Xiang, Hui Wang

**Affiliations:** ^1^Shenzhen Institutes of Advanced Technology, Chinese Academy of Sciences, Shenzhen, Guangdong, China; ^2^School of Sports Medicine and Health, Chengdu Sport University, Chengdu, Sichuan, China; ^3^Bao’an District Konghai Hospital, Shenzhen, Guangdong, China

**Keywords:** neuromodulation, neuroplasticity, bibliometrics, deep brain stimulation, transcranial magnetic stimulation

## Abstract

**Background:**

Since the 1990s, neuromodulation technology has experienced rapid advancements, providing new therapeutic approaches for clinical rehabilitation in neurological disorders. The objective of this study is to utilize CiteSpace and VOSviewer to investigate the current research status, key topics, and future trends in the field of neuromodulation technology over the past decade.

**Methods:**

Relevant literature in the field of neuromodulation technology published in Web of Science database from January 1, 2014 to June 18, 2024 were retrieved, and imported into CiteSpace and VOSviewer for visualization. VOSviewer was used for counties, institutions, authors and keywords analyses. CiteSpace was used for presentation visualization analysis of co-cited references, keywords clusters and bursts.

**Results:**

This study encompasses a total of 1,348 relevant publications, with the number of publications showing an increasing trend year by year. The most significant growth was observed between 2020 and 2021. The United States, China and the United Kingdom are the three leading countries with high output in this regard. The top three institutions in terms of the publication volume are Harvard Medical School, the University of Toronto and Stanford University. Keyword co-occurrence and cluster analysis identified that deep brain stimulation, transcranial magnetic stimulation, transcranial direct current stimulation, and focused ultrasound stimulation are the most widely used central nerve stimulation techniques in neuromodulation. The treatment of intractable chronic pain also emerged as a key focus within neuromodulation techniques. The recent keywords bursts included terms such as recovery, movement, nucleus, modeling and plasticity, suggesting that the future research trend will be centered on these areas.

**Conclusion:**

In conclusion, neuromodulation technology is garnering increasing attention from researchers and is currently widely used in brain diseases. Future research is expected to delve deeper, particularly into exploring deep brain structure stimulation targets and restoring motor function based on neuroplasticity theory.

## 1 Introduction

Neuromodulation technology refers to “the alteration of nerve activity through targeted delivery of a stimulus, such as electrical stimulation or chemical agents, to specific neurological sites in the body.”^1^ By influencing the conduction of neural signals, this technology aims to improve patients’ neurological functions and enhance their quality of life (Liu et al., 2024). It can be categorized into invasive methods, like deep brain stimulation (DBS), and non-invasive techniques, including transcranial direct current stimulation (tDCS), transcranial magnetic stimulation (TMS), and transcranial focused ultrasound stimulation (tFUS) (Davidson et al., 2024).

Since the 1990s, neuromodulation technology has experienced advancements, offering new therapeutic approaches for clinical rehabilitation in neurological disorders. This technology is now applied in the clinical treatment of diseases such as Alzheimer’s disease, stroke, and Parkinson’s disease (PD), with promising therapeutic outcomes (Chung et al., 2020; Keser et al., 2023; Shinzato et al., 2024). Compared to traditional rehabilitation therapies, the advantages of neuromodulation lie in its intelligence and precision, aligning closely with the evolving needs of global rehabilitation medicine (Li et al., 2023). Therefore, summarizing the current research status of neuromodulation technology is instrumental for researchers to elevate their work in this field and to better plan for the future development of neuromodulation technology.

In this study, VOSviewer and CiteSpacewe were utilized to visualize and analyze the research landscape of neuromodulation-related studies indexed in the Web of Science database from 2014 to 2024. VOSviewer specializes in constructing and visualizing bibliometric networks, enabling the examination of co-authorship, citation, and co-occurrence patterns. CiteSpace, on the other hand, a visualization analysis software developed by Chen (2004), features citation networks, co-citation analyses, and thematic evolution pathways. It has been widely used in academic research, knowledge management, and technological innovation. Both tools provide robust frameworks for understanding the evolution and structure of research domains, enhancing the interpretability of complex bibliometric data. This study aims to explore international research hotspots and development trends using CiteSpace and VOSviewer, thereby providing valuable insights for the advancement of neuromodulation technology research.

## 2 Materials and methods

### 2.1 Data source and search strategy

A search was conducted within the Core Collection of the Web of Science database for the time period from January 1, 2014, to June 18, 2024. The search query included: “TS = (non-invasive neuromodulation) OR TS = (invasive neuromodulation) OR TS = (neuromodulation technology).” Studies were selected based on the following inclusion criteria:

(1) Literature relevant to neuromodulation technology, encompassing both non-invasive and invasive techniques.
(2) Clinical trials, reviews, meta-analyses, observational studies, systematic evaluations, and animal experiments.
(3) Articles published in English.

Exclusion criteria comprised:

(1) Studies not aligned with the research theme.
(2) Conference abstracts, reports, news items, or documents lacking sufficient information or being duplicates.

Two researchers independently reviewed the titles, abstracts, and full texts of the retrieved studies based on the inclusion and exclusion criteria. In cases of disagreement, a third researcher would arbitrate by reviewing the contentious material, leading to a discussion that determined final inclusion. Selected studies were then exported in plain text file format and named “download_XXX.”

### 2.2 Data processing

The data were imported into CiteSpace 6.2.R4 and VOSviewer 1.6.20, with the time span set from January 2014 to December 2024. The parameters of CiteSpace were set as below: (1) the time slice was configured as 1 year per slice; (2) the system’s default link strength setting “Cosine” was used, and the node selection method was set to “Top N,” adjusting the *N*-value according to the number of nodes. The configuration of VOSviewer was established as follows: (1) the analysis type was designated as co-authorship and co-occurrence; (2) the full counting method was applied for data processing; (3) depending on the specific requirements of the data analysis, various visualization maps, including network, overlay, and density visualizations, were selected. VOSviewer was used for counties, institutions, authors and keywords analyses. CiteSpace was used for presentation visualization analysis of co-cited references, keywords clusters and bursts.

## 3 Results

### 3.1 Publication trends analysis

**FIGURE 1:**
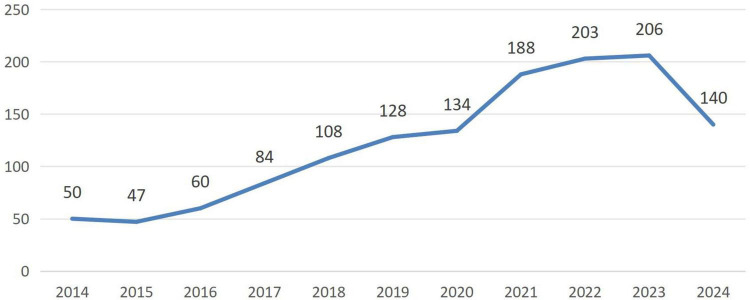
Trend in the number of articles published on neuromodulation technology research in the last decade.

A total of 1,424 articles were initially retrieved. Ultimately, 1,348 articles were included for visualization analysis after screening titles and abstracts to exclude studies unrelated to neuromodulation technology. The average number of publications per year from 2014 to 2023 was 120.8. Compared to 2014, the number of publications decreased by three in 2015 but showed a steady increase each subsequent year until 2023. Notably, the most significant growth occurred between 2020 and 2021. By 2023, the publication volume had reached approximately four times that of 2014 (Figure 1). Overall, there has been an increasing interest among international researchers in neuromodulation technology.

### 3.2 Countries/regions analysis

**FIGURE 2:**
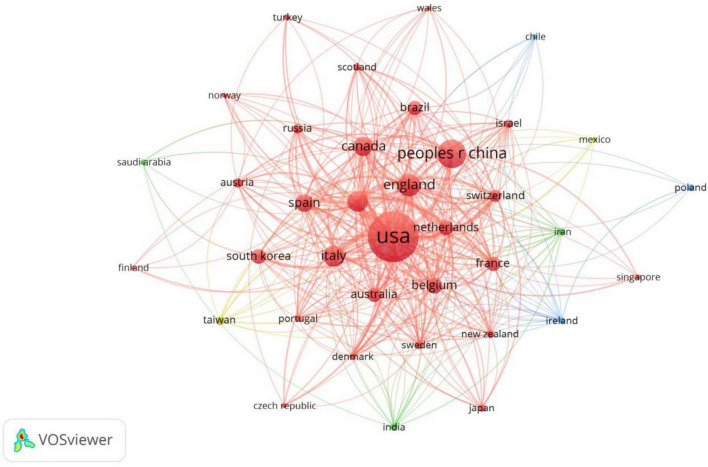
Mapping of country/region partnerships. The node size represents the frequency of publications, while the connecting lines signify international collaborations. The larger nodes for the United States, China, and the United Kingdom indicate a higher volume of publications. Collaboration among European and American countries is notably strong, whereas participation in international cooperation by Asian countries remains relatively limited.

A total of 66 countries are involved in neuromodulation technology, and Table 1 displays the top 10 countries/regions in terms of frequency, with the top three being the United States (631 publications), China (206 publications), and the United Kingdom (127 publications). From the perspective of international collaboration (Figure 2), the connections between the United States, China, and European countries (such as the United Kingdom, Germany, and the Netherlands) are notably dense, indicating frequent scientific collaboration among these nations and the formation of a relatively tight cooperative network. In contrast, some countries (such as Brazil and Chile) exhibit smaller nodes and fewer connecting lines, suggesting limited research scale and international collaboration in this field. Overall, research in neuromodulation technology demonstrates a pattern dominated by European and American countries with close international cooperation, while China, as an emerging research force, is rapidly rising and actively participating in the global collaborative network.

**TABLE 1:** Top 10 countries/areas in terms of number of publications.

Rank	Country	Publications
1	America	631
2	China	206
3	England	127
4	Italy	107
5	Germany	105
6	Canada	93
7	Spain	79
8	Belgium	63
9	Netherlands	54
10	South Korea	54

### 3.3 Institutions

**FIGURE 3:**
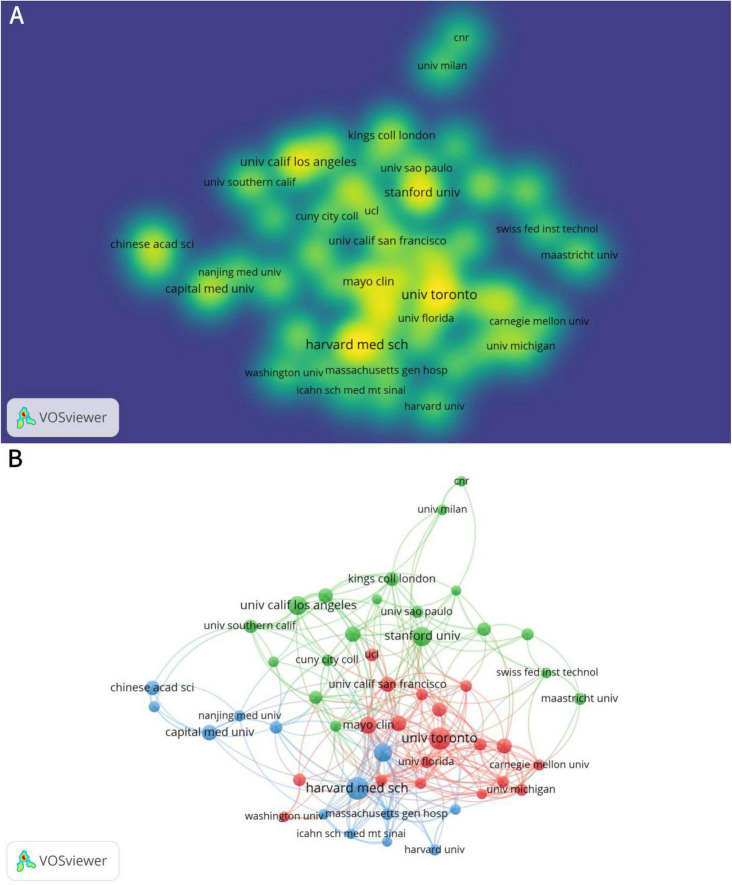
Analysis of institutions in the field of neuromodulation technology. (A) Density visualization of institutions. The density reflects the volume of publications, with institutions such as the University of Toronto, Mayo Clinic, Harvard Medical School, and Stanford University exhibiting higher publication outputs. (B) The network visualization diagram of institutional cooperation. The connecting lines between nodes represent collaborations among different institutions, with a predominant focus on partnerships between universities in Europe and North America. Nodes of the same color indicate a higher similarity in collaborative networks or research themes.

There are 2,168 institutions involved in the study of neuromodulation technology. The institution with the highest number of publications is the Harvard Medical School, which published 48 articles, followed by University of Toronto (46 articles), and then the Stanford University (35 articles) (Table 2). Capital Medical University ranks 7th in terms of publication volume (23 articles), standing as the only Asian institution among the top contributors. Figure 3A provides a more intuitive visualization of the publication output across major universities. Figure 3B illustrates that research in neuromodulation technology is predominantly led by top-tier American universities, with close international collaboration. Notably, Harvard University, the University of California system, and Stanford University are densely connected, indicating strong collaborative ties. Additionally, the University of Toronto and the University of Oxford also exhibit significant collaboration with leading American institutions.

**TABLE 2:** Top 10 institutions in terms of number of publications.

Rank	Institution	Publications
1	Harvard Medical School	48
2	University of Toronto	46
3	Stanford University	35
4	University of California, Los Angeles (UCLA)	34
5	University of Minnesota	31
6	Mayo Clinic	27
7	Capital Medical University	23
8	University of Oxford	23
9	Columbia University	22
10	University of California, San Francisco (UCSF)	21

### 3.4 Authors

**FIGURE 4:**
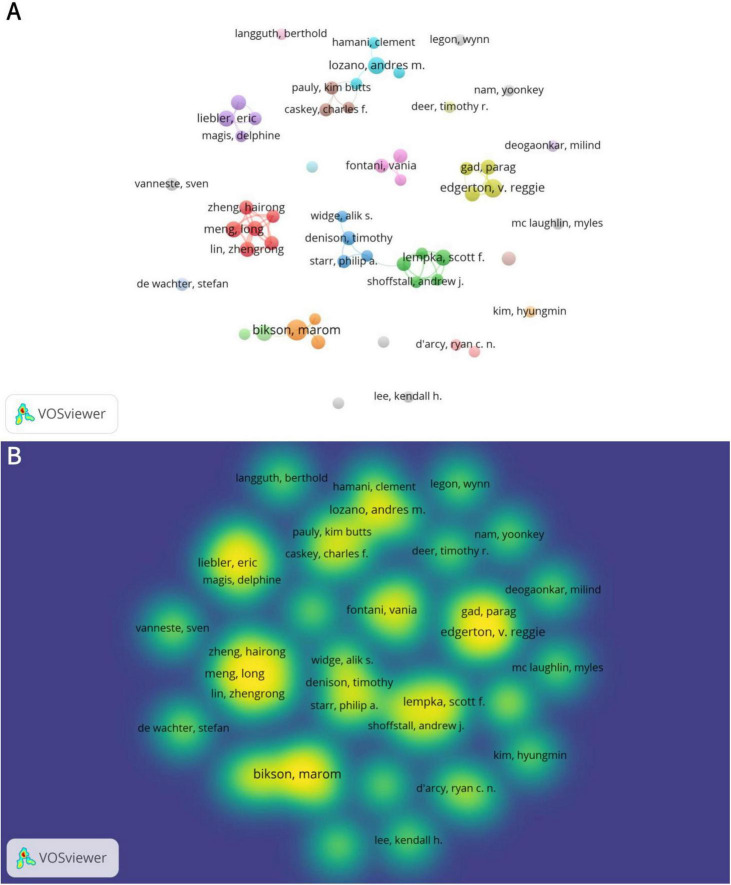
The collaboration of authors in the field of neuromodulation technology. (A) Co-occurrence map of authors. The size of the nodes represents the publication volume of authors, with larger nodes indicating higher output. The connecting lines denote collaborations between authors, and nodes of the same color signify alignment in research directions as well as stronger collaborative relationships. (B) Density visualization of authors. The density reflects the publication volume of authors, with higher density areas indicating closer collaborative relationships among authors within the same region. The intersections between different density areas highlight the exchange and cooperation among research teams, institutions, or countries.

A total of 7,673 authors have published articles related to neuromodulation. Table 3 summarizes the top 10 authors by publication volume. Marom Bikson from The City College of New York leads with 15 articles, followed by V. Reggie Edgerton (12 articles) and Andres M. Lozano (10 articles). High-productivity authors primarily collaborate within the same country or institution (Figure 4A). For instance, Hairong Zheng, Long Meng, and Zhengrong Lin are all affiliated with institutions in China, while Lozano, Andres M. and Hamani, Clement are from the University of Toronto. This collaborative pattern may be attributed to geographical proximity, shared institutional support, and similar research policy environments. Figure 4B also illustrates the publication output and collaborative relationships among authors.

**TABLE 3:** Top 10 authors in terms of publications.

Rank	Author	Publications
1	Bikson, Marom	15
2	Edgerton, V Reggie	12
3	Lozano, Andres M	10
4	Fregni, Felipe	9
5	Lempka, Scott F	9
6	Liebler, Eric	9
7	Meng, Long	9
8	Gad, Parag	8
9	Goadsby, Peter J	8
10	Lin, Zhengrong	8

### 3.5 Co-cited literature

**FIGURE 5:**
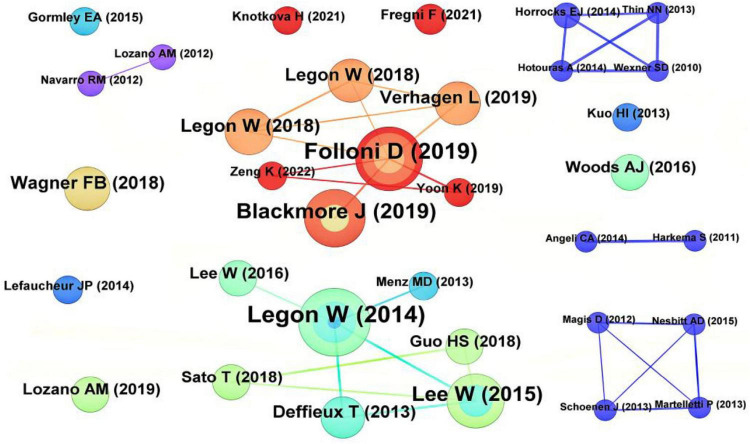
Co-cited references analysis map. The size of the nodes represents the citation frequency, with larger nodes indicating higher citation counts. The connecting lines denote co-citation relationships between two references, and the thickness of the lines reflects the frequency of co-citation.

Highly cited literature was mostly published before 2020 (Table 4), possibly due to fewer high- citation frequency, proving that tFUS can locally modulate human sensory-evoked brain activity and cortical function (Legon et al., 2014). Folloni et al.’s study on using tFUS to manipulate subcortical and deep cortical activities in primates ranked second in citation frequency (Folloni et al., 2019). Blackmore et al.’s review article on ultrasound neuromodulation ranked third (Blackmore et al., 2019). It is evident that the top 3 cited literatures are related to ultrasound neuromodulation (Figure 5).

**TABLE 4:** The top 10 co-citation frequency literature.

Rank	Study	Citation
1	Legon et al. (2014)	30
2	Folloni et al. (2019)	26
3	Blackmore et al. (2019)	21
4	Lee et al. (2015)	19
5	Legon et al. (2018b)	13
6	Verhagen et al. (2019)	12
7	Wagner et al. (2018)	12
8	Legon et al. (2018a)	12
9	Deffieux et al. (2013)	11
10	Guo et al. (2018)	8

### 3.6 Keywords

#### 3.6.1 Keywords co-occurrence analysis

**FIGURE 6:**
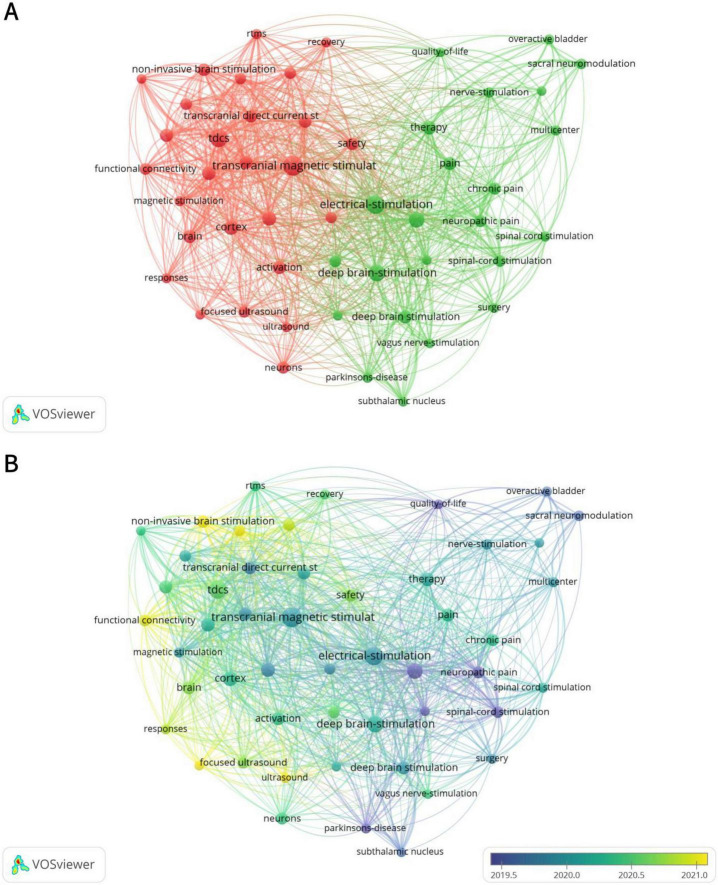
Analysis of keywords. (A) Co-occurrence map of keywords. The size of the nodes indicates the frequency of keyword occurrence, with larger nodes representing higher frequencies. The two distinct colors reflect different research focuses. (B) The time-overlay map of the cooperation network among the authors. Nodes of different colors correspond to the timeline, representing research hotspots in different years.

Excluding keywords related to search strategies, there were 4 keywords with a frequency ≥ 100: deep brain stimulation (170 times), transcranial magnetic stimulation (149 times), electrical stimulation (124 times), and tdcs (117 times) (Table 5). Due to the presence of two spelling variations, namely “deep brain stimulation” (104 occurrences) and “deep brain-stimulation” (66 occurrences), the combined frequency of DBS reaches 170, making it the most frequently mentioned term. Figure 6A illustrates 2 distinct clusters. The red cluster is primarily centered on non-invasive brain stimulation techniques, including TMS, tDCS, and FUS, with a focus on their applications in functional connectivity and recovery. The green cluster encompasses DBS and electrical stimulation, highlighting their role in pain management. This suggests that the field’s research emphasis is on modulating brain activity through non-invasive approaches to enhance neural function, while also demonstrating significant progress in addressing chronic pain and neuropathic pain. Figure 6B further reveals a shift in research trends: prior to 2020, the primary research hotspots were PD, neuropathic pain, and spinal cord stimulation (SCS), whereas post-2020, non-invasive brain stimulation has emerged as the dominant area of interest.

**TABLE 5:** Co-occurrence of top 10 keywords.

Rank	Keywords	Frequency
1	Deep brain-stimulation	170
2	Transcranial magnetic stimulation	149
3	Electrical-stimulation	124
4	tDCS	117
5	Neurostimulation	90
6	Cortex	78
7	Brain-stimulation	76
8	Therapy	70
9	Excitability	67
10	Activation	66

#### 3.6.2 Keywords clustering analysis

**FIGURE 7:**
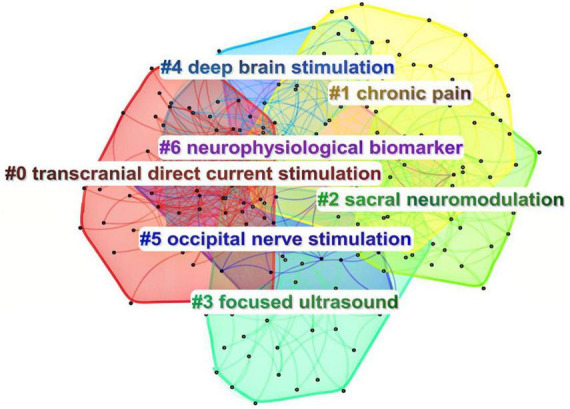
Clustering map of keywords. Different colors represent different clusters.

The log-likelihood ratio algorithm in CiteSpace software was employed to conduct keyword clustering analysis. This process generated seven cluster labels, including: #0 Transcranial Direct Current Stimulation, #1 Chronic Pain, #2 Sacral Neuromodulation, #3 Focused Ultrasound, #4 Deep Brain Stimulation, #5 Occipital Nerve Stimulation, and #6 Neurophysiological Biomarkers (Table 6). The quality of the clustering is measured by the modularity value (*Q*-value), while the average silhouette value (*S*-value) assesses the quality of the cluster structure. A *Q*-value greater than 0.3 indicates significant clustering, and an *S*-value greater than 0.7 suggests high clustering efficiency. The clustering analysis yielded a *Q*-value of 0.437 and an *S*-value of 0.7526, demonstrating both significant clustering and high-quality structure (Figure 7).

**TABLE 6:** Keyword clustering.

Cluster-ID	Size	Silhouette	Cluster labels (LLR)
#0	55	0.785	Transcranial direct current stimulation; attentional bias; social anxiety disorder; attention bias modification; corticospinal excitability
#1	40	0.759	Chronic pain; spinal cord stimulation; complex regional pain syndrome; dorsal root ganglion stimulation; incremental cost-effectiveness ratio
#2	36	0.81	Overactive bladder; urge incontinence; tibial nerve; urgency frequency; clinical success
#3	35	0.616	Focused ultrasound; non-human primates; neuron modeling; corticospinal excitability; therapeutic ultrasound
#4	32	0.745	Deep brain stimulation; local field potentials; phase-amplitude coupling; structural mri; functional mri
#5	13	0.831	Occipital nerve stimulation; cluster headache; chronic migraine; chronic headache; occipital neuralgia
#6	12	0.745	Transcranial magnetic stimulation; transcranial direct current stimulation; non-invasive brain stimulation; working memory; cognitive enhancement

#### 3.6.3 Burst detection analysis of keywords

**FIGURE 8:**
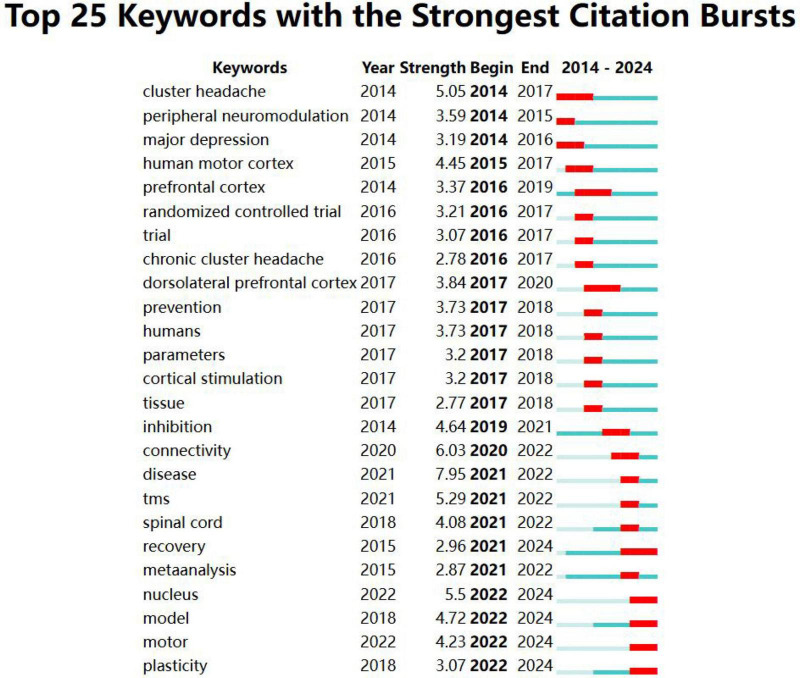
Mapping of bursting keywords. “Year” refers to the time when the keyword first appeared, “Begin” indicates the start of the burst period, and “End” denotes the conclusion of the burst. On the timeline corresponding to each keyword, the light blue phase represents the period when the keyword had not yet emerged, while the blue and red phases indicate the period when the keyword began to be cited. Additionally, the red phase signifies that the keyword was highly cited during this time.

Burst detection analysis was conducted using CiteSpace software (Figure 8). In the field of neuromodulation technology, the earliest bursting keywords were cluster headache, peripheral neuromodulation, and major depressive disorder. In contrast, model, movement, and plasticity emerged as bursting keywords in the past 3 years. The top five keywords with the highest burst strength are disease, connectivity, nucleus, TMS, and cluster headache. Notably, the keyword plasticity appeared in 2018 and has been bursting since 2022, continuing up to the present. This suggests that neural plasticity is likely to remain a focal point for international researchers in the future.

## 4 Discussion

### 4.1 Current state of research

Over the past decade, there has been a steady increase in publications within the field of neuromodulation technology, with a significant surge between 2020 and 2023. As of June 2024, 140 articles have already been published, suggesting that this year will see an even greater increase in publication volume. This trend underscores the high research value of this topic. Neuromodulation, as an emerging biotechnology, is now widely used for treating brain disorders, epilepsy, motor dysfunction, psychiatric conditions, and addiction (Zhang et al., 2020; Caulfield et al., 2022; Tervo et al., 2022). The continuous optimization of neuromodulation devices, exploration of more precise stimulation targets for different diseases, and interdisciplinary integration are enhancing the precision, procedural nature, and personalization of clinical treatments, thereby improving patient compliance and therapeutic outcomes.

From the perspective of publication volumes by country, only China and South Korea represent Asia among the top 10 countries, while the United States leads in publication volume, forming a closely-knit collaboration network with other high-impact countries. High-productivity institutions such as Harvard Medical School and the University of Toronto also lead in publication volume and influence.

### 4.2 Research hotspots

Keyword co-occurrence analysis reveals that the most frequent keyword in the field of neuromodulation in the past 10 years is DBS. DBS is a technique that modulates neural circuits by implanting electrodes at specific targets deep in the brain in order to improve brain function (Okun, 2012). Recent advancements in DBS have highlighted the potential of data-driven adaptive DBS systems, as reported by Oehrn et al. These systems utilize subthalamic nucleus or cortical field potentials to automatically adjust stimulation parameters, demonstrating superior efficacy in improving motor function and quality of life in PD patients compared to traditional DBS (Oehrn et al., 2024). The success of DBS in PD has expanded its application to other conditions, such as drug-resistant epilepsy and treatment-refractory depression, where it has shown significant benefits in reducing seizure frequency and alleviating depressive symptoms compared to sham stimulation or baseline conditions (Bewernick et al., 2017; Salanova et al., 2021). Additionally, the integration of intraoperative magnetic resonance imaging (MRI) for guiding and validating lead placement in DBS procedures has enhanced precision and safety, with targeting errors of less than 1 mm and a postoperative hemorrhage rate of only 0.6% (Rajabian et al., 2023). In a novel approach, Vassiliadis et al. introduced transcranial temporal interference stimulation (tTIS), a technique that combines striatal stimulation with electric field modeling, behavioral analysis, and functional MRI (fMRI) to investigate the causal role of the striatum in motor skill reinforcement learning in healthy subjects. Their findings suggest that this technique enhances the striatum’s neuromodulatory effects on frontal cortical regions involved in reinforcement motor learning, which can non-invasively and selectively stimulate deep brain structures and is well tolerated by patients (Vassiliadis et al., 2024). In recent years, more than 200,000 DBS implantable devices have been used worldwide to treat brain disorders (Vedam-Mai et al., 2021). A DBS remote programmed control system has been created in China, which realizes safe, timely and effective remote DBS parameter adjustments through the new Bluetooth technology, and this system was notably applied during the COVID-19 pandemic (Zhang et al., 2020). The second most frequent keyword is TMS, a non-invasive neuromodulation technique. While it offers higher safety compared to the traditional DBS, it has a poorer penetration ability and spatial resolution, and is unable to precisely locate deep brain areas (Tufail et al., 2010).

Caulfield et al. introduced an innovative neuronavigation technique capable of synchronizing the display of a patient’s head with its position on MRI scans, offering real-time feedback to researchers during localization. Compared to traditional marker-based localization performed by trained TMS operators, this neuronavigation-based approach demonstrated significantly greater precision in positioning the TMS coil, with reduced deviations in distance, pitch angle, and yaw angle relative to the scalp target (Caulfield et al., 2022). In addition, coupling TMS with Electroencephalogram (EEG), by analyzing TMS-induced EEG signals in real time, it is possible to determine the stimulation target of TMS and simultaneously adjust the stimulation parameters to produce the desired stimulation intensity at the level of the target cortex and achieve a satisfactory therapeutic effect (Tervo et al., 2022). The third most frequent keyword is electrical stimulation, which includes techniques such as tDCS and vagus nerve stimulation (VNS) in addition to DBS. TDCS has become more prevalent in recent years, with traditional tDCS using two larger spacer electrodes that are unable to stimulate the target more centrally (Parlikar et al., 2021). In contrast, high-definition tDCS surrounds the central electrode with four return electrodes (Müller et al., 2022), isolating the stimulated area to achieve more precise target stimulation and longer-lasting stimulation effects (Parlikar et al., 2021). VNS is primarily used to treat brain disorders like neurodegenerative diseases, cerebrovascular diseases, and psychiatric disorders (Wang et al., 2021). While it is generally well tolerated by patients, the adverse effects of implanted stimulation devices, including surgical risks and battery replacement concerns, should not be overlooked (Dawson et al., 2021). Transcutaneous cervical VNS (tcVNS) and transcutaneous auricular VNS (taVNS) offer non-invasive alternatives that avoid risks associated with surgical implantation and battery replacement. However, tcVNS requires a higher threshold to activate the Hering-Breuer reflex compared to implanted cuff electrode stimulation, while taVNS fails to activate the reflex under any parameters, indicating limited efficacy in activating A-fibers of the vagus nerve (Bucksot et al., 2020). A highly cited review discusses the emerging concept of closed-loop transcranial electrical stimulation integrated with fMRI. This approach requires subjects to modulate brain activity according to specific instructions to engage targeted neural regions. Blood oxygenation level dependent signals are analyzed algorithmically to visualize target engagement levels, and dynamic functional connectivity is measured using Fisher’s z. The extracted metrics are compared and fed into an optimization algorithm to determine optimal stimulation parameters. Real-time feedback is then used to update the stimulation device with the next set of optimal parameters until predefined stopping criteria are met. This method enables rapid adaptation to other brain stimulation protocols (Soleimani et al., 2023). In summary, achieving more precise targeting in non-invasive neuromodulation continues to be a prominent research focus. Technological advancements, including high-definition tDCS and remote DBS systems, are refining stimulation techniques to minimize or eliminate surgical risks, thereby enhancing treatment efficacy and expanding clinical applicability. Additionally, integrating brain physiological signals to enable closed-loop, personalized stimulation parameter adjustments represents a significant future research direction.

Co-cited literature is an important indicator of research hotspots within a specific field, offering insights into the most recognized studies in neuromodulation technology. Analyzing the high-frequency co-cited literature in recent years helps identify the emerging research directions in this field. Notably, research on FUS dominates the top 10 co-cited frequency literature. Legon et al. applied tFUS and TMS simultaneously and coaxially to the human primary motor cortex. This study non-invasively detected the effect of ultrasound on the excitability of motor neurons through motor evoked potentials. They found that ultrasound causes a single-pulse amplitude and intracortical facilitation of motor evoked potentials but not attenuate intracortical inhibition, as well as increasing response speed and shortening reaction time to a simple stimulus response task, reporting for the first time the effects of ultrasound on the excitability and motor performance of the human motor cortex (Legon et al., 2018b). Verhagen et al. focused on the tFUS stimulation. They observed region-specific tFUS effects after a 40-s period of tFUS stimulation, particularly in two medial frontal brain regions (supplementary motor area and frontal polar cortex) of macaques. Interestingly, tFUS also induced signaling changes in the meningeal septum and lasted for almost 2 h (Verhagen et al., 2019). By combining fMRI with tFUS, they were able to detect cortical effects with high spatial resolution (Folloni et al., 2019). This approach holds promising potential for overcoming the limitations of individual techniques, positioning it as a powerful tool for future neuroscience research.

Keyword clustering analysis showed that, in addition to tDCS, FUS and DBS (The analysis of keyword contribution maps and co-citation literature maps, as referenced earlier, is equally applicable to the cluster terms discussed in this section), other emerging research hotspots in the field of neuromodulation techniques include chronic pain, sacral neuromodulation, occipital nerve stimulation and neurophysiological biomarkers. SCS has gained attention as a promising neuromodulation technique for the treatment of chronic pain (Knotkova et al., 2021), which is recommended by clinical application guidelines published in the European Union, the United Kingdom, and the United States because of its high safety and efficacy (Ferraro et al., 2022). One particularly innovative approach is closed-loop SCS, which records spinal cord evoked compound action potentials from each stimulation pulse (Muller et al., 2024; Nijhuis et al., 2024). Studies have shown that closed-loop SCS utilizing spinal evoked compound action potential control is more effective in relieving chronic low back pain than fixed-output SCS. This effect is maintained with a 2-year follow-up, with the closed-loop group exhibiting significantly higher spinal cord activation during the treatment window (Mekhail et al., 2022). At the same time, a large number of studies have also used non-invasive brain stimulation for analgesic effects. Todd et al. investigated the application of FUS in treating chronic pain related to the central nervous system. They highlighted FUS as a promising emerging technology for neuromodulation-based pain management. However, the authors noted that its clinical translation remains in the early stages, with only a limited number of ablation studies targeting pain. Additionally, areas such as blood-brain barrier opening and neuromodulation have been largely unexplored in this context (Todd et al., 2020). Molero-Chamizo et al. applied bilateral hemispheric tDCS to address limb pain and spasticity in stroke patients. Their findings demonstrated a significant reduction in upper limb pain and a marked improvement in spasticity compared to pre-intervention levels (Molero-Chamizo et al., 2021). In the area of sacral neuromodulation, this technique is mostly used for the treatment of lower urinary tract dysfunction (Meng et al., 2024). A hot research topic in this field involves comparing the safety, efficacy, and subjective patient satisfaction between two treatments, sacral neuromodulation and botulinum toxin A (Amundsen et al., 2018). Occipital nerve stimulation is another research hotspots in the field of neuromodulation, mainly for the treatment of chronic refractory cluster headache (Brandt et al., 2023; Membrilla et al., 2023). Looking ahead, studies utilizing imaging or electrophysiology to guide neuromodulation and identifying audience populations based on biomarker-driven approaches are key to further advancing the translation of neuromodulation technologies into clinical practice (Keser et al., 2023).

### 4.3 Research trends

Keyword emergence reflects the increasing citation frequency of specific terms over time, indicating the research frontiers and trends in the field. Recent emerging keywords such as recovery, nucleus accumbens, modeling, motor and plasticity suggest that the future research trend in this field may focus on the application of neuromodulation techniques to enhance brain plasticity for motor function recovery. One promising direction is the stimulation of the corticobasal ganglia system using non-invasive or invasive neuromodulation to modulate the imbalance between long-term potentiation and long-term depression (Bove et al., 2024). Bao et al. localized primary motor cortex circuits at approximately 5 and 16 mm from the cortical surface according to magnetic resonance tomography images of patients, and applied continuous theta burst transcranial ultrasound to stimulate the circuits (Bao et al., 2024). With the ongoing progress of brain science, the target of neuromodulation has been explored to deep brain nuclei. For instance, tFUS stimulation of the nucleus ambiguus has been shown to effectively improve drug addiction (Peng et al., 2024).

When tFUS was applied to stimulate the caudate nucleus in non-human primates, it enhanced functional connectivity between the caudate nucleus and the insular cortex while suppressing connectivity between the caudate nucleus and the motor cortex. These findings suggest that tFUS targeting deep brain structures can modulate functional connectivity within the default mode network and the frontotemporal network (Liu et al., 2023). Vassiliadis et al. conducted 80 Hz tTIS of human striatum to investigate the brain mechanism of reinforced feedback to improve motor learning, providing an innovative tool for exploring the relationship between deep brain structures and motor learning (Vassiliadis et al., 2024). Given these advancements, it is expected that neuromodulation targeting deep brain regions will become a key focus of future research. The ability to alter functional connectivity and study the relationship between deep brain structures and motor learning offers novel opportunities for further exploration and clinical applications. Furthermore, the integration of neuromodulation techniques with rehabilitation robotics or brain-computer interfaces to address motor dysfunction represents a promising direction for future research. Juan et al. combined bilateral tDCS with end-effector robotic-assisted rehabilitation, where stroke patients received tDCS while performing robot-assisted upper limb exercises. After 20 sessions, patients undergoing the combined therapy showed no significant difference in gross motor function of the upper limb compared to those receiving robotic therapy alone. However, the combined approach demonstrated superior improvements in finger flexion function (Bernal-Jiménez et al., 2024).

### 4.4 Limitations

The vast amount of neuromodulation-related literature poses a challenge, and the search strategy employed in this study has resulted in the exclusion of some relevant papers. Through search and comparison, we found that while this paper could provide a comprehensive overview of the major hotspots and trends in the field of neuromodulation technology, it still may omit certain technologies such as optogenetics. This paper only includes the core database of Web of Science, and it does not include other databases, such as PubMed. Furthermore, the lack of a standardized process for the visualization and analysis of CiteSpace, such as the absence of time slicing and thresholding, may lead to a certain degree of bias in the final data.

## 5 Conclusion

The field of neuromodulation techniques has advanced rapidly in the last decade. Key techniques include brain stimulation techniques such as DBS, TMS, tDCS, and tFUS, as well as peripheral nerve stimulation techniques such as sacral neuromodulation, and occipital nerve stimulation. The integration of imaging technologies to improve the safety and localization accuracy of invasive or non-invasive neuromodulation techniques has become a hot topic. Moving forward, neuromodulation techniques are expected to focus on the exploration of deep brain stimulation targets and the restoration of brain function based on the theory of neuroplasticity to address movement disorders.

## Footnotes

1
www.neuromodulation.com
